# Child Consumption of Whole Fruit and Fruit Juice Following Six Months of Exposure to a Pediatric Fruit and Vegetable Prescription Program

**DOI:** 10.3390/nu12010025

**Published:** 2019-12-20

**Authors:** Amy Saxe-Custack, Jenny LaChance, Mona Hanna-Attisha

**Affiliations:** 1 Department of Food Sciences and Human Nutrition, Division of Public Health, Michigan State University–Hurley Children’s Hospital Pediatric Public Health Initiative, 200 E 1st St, Flint, MI 48502, USA; 2 Division of Public Health, Michigan State University–Hurley Children’s Hospital Pediatric Public Health Initiative, 200 E 1st St, Flint, MI 48502, USA; jlachan1@hurleymc.com; 3 Department of Pediatrics and Human Development, Division of Public Health, Michigan State University–Hurley Children’s Hospital Pediatric Public Health Initiative, 200 E 1st St, Flint, MI 48502, USA; hannamon@msu.edu

**Keywords:** fruit, fruit juice, child nutrition, dietary intake, fruit and vegetable prescriptions

## Abstract

Public health recommendations suggest limiting child consumption of fruit juice in favor of whole fruit due to juice’s high sugar content, lack of fruit fiber, and potential for excess intake. However, replacing juice with whole fruit may be particularly challenging for low-income and minority children, who report the highest intake of 100% juice. To address access and affordability challenges among low-income children, researchers partnered with pediatricians in an urban food desert community, to introduce a fruit and vegetable prescription program (FVPP) that provided a $15 prescription for fresh produce to every child during each office visit. Participating vendors included a farmers’ market and local mobile market. This study assessed changes in daily consumption of total fruit and whole fruit among 108 pediatric patients following six months of exposure to the FVPP. Child-reported mean daily intake of whole fruit increased significantly from the baseline to the 6-month follow-up (*p* = 0.03): 44% of children reported an increased intake of at least ¼ cup per day, and 30% reported an increased intake of at least ½ cup per day. Changes in total fruit intake (including fruit juice) were not significant. Results suggest a pediatric FVPP may have meaningful impacts on children’s dietary behaviors, particularly with regard to the intake of whole fruits.

## 1. Introduction

Although consumption of fruit has been associated with a reduced risk of obesity and chronic disease [1,2,3,4,5,6], better psychological well-being [1,7,8,9], and improved gut health [1,10], intake fails to meet dietary recommendations among all but the youngest children in the US [11,12]. Considering the growing evidence of an association between greater fruit and vegetable consumption during childhood and reduced chronic diseases in adulthood [13,14,15,16], both short- and long-term implications of programs that successfully increase fruit intake among children are likely to be significant. However, public health initiatives should not only address deficits in nutrition knowledge [17,18], but also enduring challenges with regard to the access to and affordability of fresh, high-quality fruits—particularly among low-income children [6,19,20,21].

When considering intake of total fruit, which includes 100% fruit juice, it is important to note that fruit juice, which is often less expensive and more accessible, does not necessarily carry the same benefits of whole fruit [4]. Although 100% fruit juice is a good source of important nutrients, such as vitamin C, folate, and potassium [22,23], its high sugar content, lack of fruit fiber, and potential for excess intake are concerning [24,25]. Accordingly, recommendations from the American Academy of Pediatrics (AAP) suggest limiting fruit juice consumption to no more than 8 fluid ounces for children 7 to 18 years of age [22]. Additionally, the AAP as well as the Dietary Guidelines for Americans recommend that children be encouraged to consume whole fruit in place of juice [25,26]. The advice to replace 100% juice with whole fruit may be especially challenging for low-income children throughout the US, who are less likely to consume adequate amounts of whole fruit than their higher-income peers [11,24,27,28,29]. To specifically address barriers to accessing fresh produce, some healthcare practices have introduced fruit and vegetable prescriptions that are written by physicians to exchange for fresh produce [30,31,32,33]. Thus far, no studies have examined whether exposure to pediatric fruit and vegetable prescription programs influence consumption of whole fruit among children.

Pediatricians care for individuals from infancy to early adulthood, and their influence on dietary behaviors during this period is likely to have long-term health, growth, and developmental impacts [34,35,36,37]. Although challenges with food security and limited food access in children have been associated with poor diet quality [38], negative health outcomes [39,40,41], and poor academic achievement [42], pediatricians and other primary care physicians often do not address the underlying problem [43,44]. In addition to screening for food insecurity among households with children [45], pediatricians should actively promote the consumption of foods that are high in the nutrients needed to support healthy growth and development. To confront persistent barriers to the access to and affordability of fresh fruits and vegetables in a food desert community [6,19,46], researchers in the current study partnered with pediatricians to expand a successful fruit and vegetable prescription program, which supplied every child with a $15 prescription for fresh produce during each office visit. Participating vendors included the downtown farmers’ market and a local mobile market. The purpose of this study was to examine changes in the consumption of whole fruit among a sample of children (7–18 years of age) following six months of exposure to the pediatric fruit and vegetable prescription program.

## 2. Materials and Methods

### 2.1. Study Population

Enduring challenges with child poverty [47] and inadequate grocery stores [48] in Flint, Michigan, have been compounded further by a lead-in-water crisis that has serious health consequences for children [49,50]. In August 2018, a successful fruit and vegetable prescription program that had been initiated at a large pediatric clinic co-located with a farmers’ market was expanded to another Flint clinic to test replicability and preliminary effectiveness. The second site, located several miles away from the farmers’ market, serves approximately 3000 pediatric patients, the majority of whom live in Flint and receive public health insurance.

### 2.2. Study Design

This was a non-controlled longitudinal intervention trial with a consecutive sample of caregiver–child dyads newly exposed to a pediatric fruit and vegetable prescription program. Self-reported data was collected from caregiver–child dyads at baseline and at the 6-month follow-up. Study recruitment and the prescription program are described in full in an earlier article sharing baseline food security and dietary patterns in a subsample exclusively of Flint children [30]. All methods were approved by the Michigan State University Institutional Review Board (Study 00000666, titled “Fruit and Vegetable Prescription Program”), and conducted in accordance with the Declaration of Helsinki.

### 2.3. Pediatric Fruit and Vegetable Prescription Program

The pediatric fruit and vegetable prescriptions in the current study, similar to medical prescriptions, were written by pediatricians and given to patients. All patients received a $15 fruit and vegetable prescription at the conclusion of every clinic visit to be redeemed at either the Flint Farmers’ Market or a local mobile market. Vendors treated prescriptions as vouchers that could be redeemed only for fresh fruits and vegetables.

### 2.4. Participants and Data Collection

The fruit and vegetable prescription program was introduced to all clinic patients (0–18 years of age) in August 2018. At that time, caregivers with children who were 7 to 18 years old and spoke English were invited to participate in the research study. Caregiver–child dyads consisted of one caregiver and one child from each family, who separately completed demographic questions followed by survey questions used to assess dietary patterns, participation in food assistance programs, and food security. At approximately the 6-month follow-up (6 months after baseline data collection), caregiver–child dyads completed the identical assessments via caregiver-selected telephone or in-person interview. All data were collected using a secure digital platform (Michigan State University Qualtrics) after receiving caregiver consent and child assent.

### 2.5. Evaluation Tools

#### 2.5.1. Fruit Consumption

Child dietary patterns were measured with the 41-item Block Kids Food Screener, a tool that has been shown to have good relative validity for children and adolescents [51]. The instrument, which specifically assessed the frequency and quantity of foods and beverages consumed in the previous week, was self-administered with the support of a trained research assistant. At the beginning of the form, children are instructed to “Remember what you had for breakfast, lunch, dinner, after school, while watching TV, at bedtime, and on the weekend” before being presented with specific foods or beverages and asked “How many days last week did you eat or drink it? How much in one day?” Intake of whole fruit was determined using the following two prompts from the screener that assessed frequency and quantity of whole fresh, frozen, or canned fruit: “Apples, bananas, or oranges” and “Any other fruit, like strawberries, grapes.” Intake of juice was determined using one item from the screener that assessed frequency and quantity of 100% fruit juice: “Real fruit juice, like orange juice, apple juice, or Mexican fruit drinks like licuados (DO NOT include soda).” Total fruit consumption was determined using a total of four items, which included the three previous prompts related to whole fruit and 100% fruit juice as well as the prompt “applesauce, fruit cocktail.” Dietary analysis, using the Block Online Analysis System, provided mean daily intake of total fruit, whole fruit, and 100% fruit juice. Intake was measured in cup equivalents (1 cup equivalent of 100% fruit juice was equal to 240 mL).

#### 2.5.2. Participation in Food Assistance Programs

Caregiver survey questions included information regarding participation in a variety of local, state, and national food assistance programs. In comparison to minimal participation in other food assistance programs, a high percentage of caregivers reported household participation in the Supplemental Nutrition Assistance Program (SNAP) (45.6%), which is the US government’s largest anti-hunger program [52], and/or child participation in free and reduced-price school meals (55.3%), which provides free or reduced-price school meals to children from low-income households. Therefore, these variables were used to assess the influence of participation in food assistance programs on changes in children’s mean daily consumption of whole fruit.

#### 2.5.3. Food Security

To measure household food insecurity and hunger, caregivers completed the US Household Food Security Module: Six-Item Short Form, developed by the National Center for Health Statistics [53]. A sample item was “In the last 12 months, did you ever eat less than you felt you should because there wasn’t enough money for food?” The sum of affirmative responses (“often”, “sometimes”, “yes”, “almost every month”, “some months but not every month”) served as the household’s raw score. Food security status was assigned according to this calculated raw score (0–1 = high/marginal food security; 2–4 = low food security; 5–6 = very low food security).

Children 12–18 years of age completed the 9-item Self-Administered Food Security Survey Module for Youth [54]. This age range was selected based on the tool demonstrating adequate internal validity for children ages 12 years and older and not being recommended for use with younger children. Questions referred to the food situation in the home during the last month, and a sample item was “Did you have to eat less because your family didn’t have enough money to buy food?” The sum of affirmative responses (“a lot” or “sometimes”) served as the respondent’s raw score. Food security status was determined by the raw score (0–1 = high/marginal food security; 2–5 = low food security; 6–9 = very low food security).

### 2.6. Statistical Analyses

Paired samples *T*-tests examined changes from baseline to 6-month follow-up in terms of mean daily intake of total fruit, whole fruit, 100% fruit juice, and vegetables (measured in cup equivalents). Independent samples *T*-tests were used to compare mean change in intake of whole fruit by child gender, child race, caregiver race, and child age, and one-way ANOVA was used to compare mean change by caregiver age and education groups. Caregiver-reported household food security and child-reported food security at 6-month follow-up were dichotomized into high/marginal or low/very low food security. Chi-square analysis was used to compare ¼ and ½ cup whole fruit change by food security category at 6-month follow-up. Independent samples *T*-tests compared mean change in whole fruit consumption by food assistance programs (SNAP and free and reduced-price school meals) as well as by caregiver-reported household food security and child-reported food security categories. All data were analyzed using SPSS statistical software (version 25, IBM Corp., Armonk, NY, USA, 2015).

## 3. Results

A total of 114 caregiver–child dyads completed baseline and 6-month assessments between August 2018 and July 2019, with 94.7% of the children (*n* = 108) completing the Block Kids Food Screener at both time points. Most of the children (mean age 12.89 ± 2.86 years, range 7–18 years) were African American (62.8%) and female (55.4%). Similarly, the majority of caregivers (mean age 39.83 ± 9.46 years, range 25–79 years) were African American (60.5%) and female (94.7%).

### 3.1. Fruit and Vegetable Consumption

Baseline and 6-month follow-up measures of mean daily servings of total fruit, whole fruit, and 100% fruit juice reported in cup equivalents were compared using paired samples *T*-tests. Mean daily servings of whole fruit increased significantly (*p* = 0.029) from baseline (0.62 ± 0.69, range 0.00–3.31) to 6-month follow-up (0.81 ± 0.64, range 0.00–3.31). Of the 108 children who completed baseline and 6-month follow-up Block Kids Food Screeners, 47 (43.5%) reported an increase in mean daily consumption of whole fruit by at least ¼ cup, and 37 children (34.3%) reported an increase in mean daily consumption of whole fruit by at least ½ cup. The mean daily intake of total fruit increased from baseline (1.35 ± 1.22, range 0.01–5.78) to 6-month follow-up (1.43 ± 0.96, range 0.00–6.26), but the change was not significant (*p* = 0.548). The mean daily intake of 100% fruit juice decreased, but the change from baseline (0.60 ± 0.67, range 0.00–2.98) to 6-month follow-up (0.53 ± 0.56, range 0.00–2.80) was not significant (*p* = 0.388). There was no statistically significant change in children’s vegetable intake from baseline (0.63 ± 0.62, range 0.02–3.72) to 6-month follow-up (0.65 ± 0.40, range 0.00–1.77; *p* = 0.820).

To examine whether change in mean daily intake of whole fruit was associated with child or caregiver characteristics, we compared mean change in whole fruit consumption by key demographic variables. There was no significant difference in mean change of whole fruit intake by child gender, race, or age category (Table 1). Similarly, there was no significant difference in mean change of whole fruit intake by caregiver race, age category, or education (Table 1).

### 3.2. Participation in Food Assistance Programs

Caregiver-reported participation in most food and nutrition assistance programs was considered low. This included low participation in the Special Supplemental Nutrition Assistance Program for Women, Infants, and Children, WIC (11.4%), which provides federal grants to states for supplemental foods, health care referrals, and nutrition education for low-income pregnant and postpartum women, and to infants and children up to 5 years of age who are at nutritional risk [55]. Similarly, caregivers reported low participation at local food sites (5.3%), including food pantries, soup kitchens, and shelters that distribute food to those who have difficulty purchasing enough to avoid hunger [56], as well as Hoophouses for Health (0.9%)—a statewide program designed to increase access to fresh, locally-grown food among low-income families [57]. However, over half of the caregivers (55.3%) reported child participation in free and reduced-price school meals, and nearly half (45.6%) reported household participation in SNAP. Using independent samples *T*-tests, we examined the difference in change in mean daily intake of whole fruit by participation in SNAP and free and reduced-price school meals. There was no significant difference in this change by participation in either SNAP or free and reduced-price school meals (Table 2).

### 3.3. Food Security

To further examine whether change in whole fruit intake was influenced by household or child food security, we examined the relationship between the food security category and the change from baseline to 6-month follow-up in daily intake of whole fruit. There was no significant difference in the mean daily intake of whole fruit between caregiver-reported household food security categories at 6 months (*p* = 0.644). Similarly, there was no significant difference in change in mean daily intake of whole fruit between child-reported food security categories at 6 months (*p* = 0.344).

**Figure 1:**
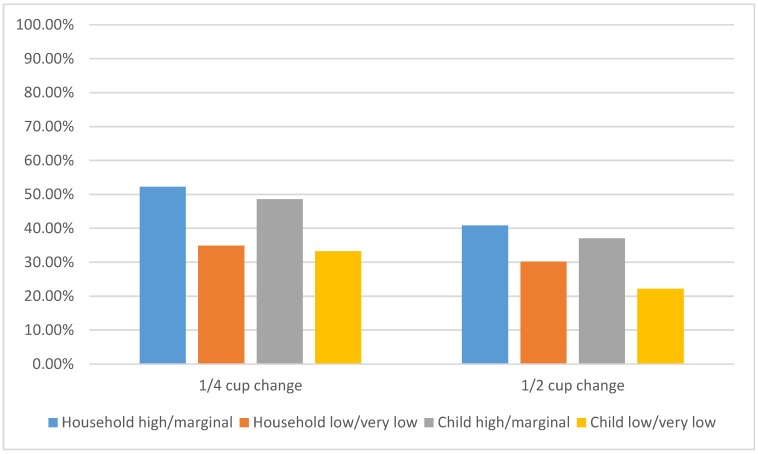
Comparison of ¼ and ½ cup whole fruit change by food security category at 6-month follow-up.

As shown in Figure 1, when considering caregiver-reported household food security measures, 23 children (52.3%) who lived in households with high/marginal food security increased daily whole fruit consumption by at least ¼ cup, compared to 15 children (34.9%) who lived in low/very low food security households (*p* = 0.102). When considering child-reported feelings of food security, 17 children (48.6%) who reported high/marginal food security increased daily whole fruit consumption by at least ¼ cup, compared to six children (33.3%) who reported low/very low food security (*p* = 0.289).

To better understand the relationship between the food security category and participation in food assistance programs, a chi-square analysis assessed the difference in the proportion of high/marginal and low/very low food security groups by participation in SNAP and school lunch programs. For households that reported high/marginal food security, 43.4% (*n* = 23) participated in SNAP, compared to 46.9% (*n* = 23) of those in the low/very food security group (*p* = 0.719). For households that reported high/marginal food security, 52.8% (*n* = 28) reported their children received free and reduced-price school meals, compared to 63.3% (*n* = 31) in the low/very food security group (*p* = 0.286). Data were similar when examining child-reported food security groups as there was no relationship between food security category and SNAP participation [high/marginal food security at 43.6% (n = 17) and low/very low at 47.8% (*n* = 11), *p* = 0.746] or free and reduced-price school meals participation [high/marginal at 59.0% (*n* = 23), low/very low at 56.5% (*n* = 13), *p* = 0.850].

## 4. Discussion

After only six months of exposure to a pediatric fruit and vegetable prescription program that provided a $15 prescription to every child at each office visit, children in our study reported a significant increase in mean daily consumption of whole fruits, without an analogous increase in consumption of fruit juice. These preliminary results suggest an important positive association between exposure to a pediatric fruit and vegetable prescription program and an increased consumption of whole fruit among children. The lack of association between exposure to the prescription program and intake of fruit juice among our sample of children is also noteworthy. In spite of recommendations to limit juice consumption [22], fruit juice contributes to approximately 34% of total fruit intake among youth 2–19 years old [58]. Although consumption of 100% fruit juice within current recommendations provides important nutrients, such as vitamin C, folate, and potassium [22,23], consistent evidence suggests that excess consumption is associated with negative health consequences among children, including increased risk of childhood obesity [59,60], dental caries [61], and type 2 diabetes [5,25,62]. Our findings suggest that pediatric fruit and vegetable prescriptions may be an effective method of increasing the intake of fresh whole fruit and fruit fiber among children, while having little to no influence on the consumption of 100% fruit juice.

The change in the intake of whole fruit was consistent across all subgroups in our sample, indicating program effectiveness regardless of child age, gender, race, or socioeconomic status. With childhood consistently identified as a critical period for the establishment of lifelong dietary patterns [35,36,37], it is particularly important to support access to healthy food and foster the development of healthy eating behaviors among all individuals early in life. The approach and design of the current study were uniquely different from the majority of programs throughout the US that utilize fruit and vegetable prescriptions as a disease-management strategy for adults with diet-related chronic health conditions [32,33,63,64,65]. Instead, pediatric fruit and vegetable prescriptions in this study were provided to all children, regardless of health condition or socioeconomic status, in an effort to emphasize the critical role of fruits and vegetables in chronic disease prevention during children’s formative years [35,36,37]. The provision of a prescription for fruits and vegetables to every child during each office visit goes beyond traditional nutrition education, to address persistent environmental challenges related to the access to and affordability of fresh produce. Furthermore, pediatricians’ provision of the prescriptions emphasized the important role that fresh, high-nutrient foods play in health promotion and disease prevention.

Consistent evidence suggests that total fruit intake is significantly lower among economically disadvantaged children and adolescents [11,24,28,66]. Although the fruit and vegetable prescription program appeared to benefit all children in our sample, the mean daily intake of whole fruit among children who lived in food-secure households increased at a slightly higher rate. It is important to note that we examined dietary changes among children after only 6 months of exposure to a new fruit and vegetable prescription program. Many caregivers had never purchased fruits or vegetables from participating vendors (Flint Farmers’ Market and the local mobile market) prior to their receipt of the prescriptions and most were entirely unfamiliar with redemption procedures. This difference in change in mean intake between children living in food-secure and food-insecure households may be at least partially explained by the short duration of exposure to the program and related follow-up data collection. Future research will examine these differences following one year of exposure to the pediatric fruit and vegetable prescription program.

Research focused on low-income adults who participate in fruit and vegetable prescription programs has reported no significant changes in produce consumption or purchasing patterns [31]. The current study, however, suggests that pediatric fruit and vegetable prescriptions have a significant impact on consumption of whole fruits, while having no influence on the consumption of fruit juice or vegetables. This finding supports previous qualitative work in Flint in which caregivers, who perceived the prescription program to have a positive influence on child consumption of fresh produce, described child preferences for fruits, particularly “new” fruits that farmers’ market vendors encouraged children to taste for the first time while using their prescriptions [6]. These findings support accumulating evidence that child participation in food selection and preparation are effective in improving diet quality [67,68,69,70]. Future research will investigate the specific influence of a cooking and nutrition program on vegetable consumption among pediatric fruit and vegetable prescription program recipients.

Finally, it has been well documented that, in comparison to higher-income neighborhoods, low-income neighborhoods have lower-quality food and fewer healthy food options [20,71]. Similarly, previous work in Flint has demonstrated that the primary barriers to fresh produce consumption among children and families are poor-quality fresh fruits and vegetables at local food stores and limited funds available to spend on food [6,46]. There is a critical need to address these persistent barriers to healthy eating during childhood and adolescence, when lifelong dietary patterns are established [35,36,72]. The fruit and vegetable prescription program has been recognized for facilitating easy access to fresh, high-quality produce, in a community with few full-service grocery stores [6,46]. Future research could examine the impact of increased fruit intake on the availability of such produce in a food desert, its impacts on interest in farming, and how concerns around water quality may impact fruit juice intake. Such research endeavors require a multidisciplinary approach to best answer these questions.

Several limitations to the current study should be noted. Our sample was small and specific to one low-income, urban community. As a result, findings may not be generalizable. Although there may have been selection bias, as responses from households that voluntarily elected to complete the survey could have differed from responses of households that did not, the study population’s characteristics strongly matched those of the source population, which largely consists of low-income, minority children receiving public health insurance. The accuracy of the Block Kids Food Screener may be limited by recall bias, but a trained research assistant was consistently available to children when completing this instrument in an effort to minimize this particular study limitation. Furthermore, this was the first study to use a dietary assessment tool, previously validated for use with children, to assess changes in dietary intake following exposure to a pediatric fruit and vegetable prescription program.

## 5. Conclusions

Pediatricians and primary care physicians are in the unique and favorable position to address the poor access and affordability of fresh, whole fruits among children. The current study suggests that after only 6 months of exposure to a pediatric fruit and vegetable prescription program, mean daily consumption of whole fruits improves significantly among children. Findings are especially important to pediatricians and primary care physicians who recognize the deleterious consequences of food insecurity and poor access among young patients [38,39,40,41,42] and are seeking tangible solutions that not only provide food to hungry children, but also ensure that the food provided is nutrient-rich to support healthy growth and development.

## Figures and Tables

**Table 1:** Comparison of change in mean daily intake of whole fruits at 6 months by key child and caregiver demographic characteristics.

	Characteristics	Mean Change ± SD	*p*-Value
Child Gender	Male (*n* = 51)	0.18 ± 0.80	0.974
	Female (*n* = 57)	0.19 ± 0.92	
Child Race	African American (*n* = 64)	0.12 ± 0.86	0.725
	White (*n* = 20)	0.19 ± 0.68	
Child Age	7–12 years (*n* = 45)	0.07 ± 0.91	0.288
	13–18 years (*n* = 50)	0.25 ± 0.72	
Caregiver Race	African American (*n* = 62)	0.10 ± 0.88	0.188
	White (*n* = 26)	0.37 ± 0.81	
Caregiver Age	25–34 years (*n* = 29)	−0.02 ± 0.94	0.149
	35–44 years (*n* = 43)	0.38 ± 0.74	
	45+ years (*n* = 24)	0.20 ± 0.86	
Caregiver Education	High school degree or less (*n* = 30)	0.29 ± 0.78	0.932
	Some college/Technical school/Associate’s degree (*n* = 41)	0.21 ± 0.88	
	Bachelor/Graduate degree (*n* = 20)	0.21 ± 0.94	

**Table 2:** Comparison of change in mean daily intake of whole fruits at 6 months by food assistance programs.

Food Assistance Program	Participation	Mean Change	*p*-Value
SNAP	Yes (*n* = 44)	0.18 ± 0.86	0.714
	No (*n* = 52)	0.24 ± 0.83	
School lunch	Yes (*n* = 57)	0.13 ± 0.91	0.284
	No (*n* = 39)	0.33 ± 0.74	
